# Kanatzidisite: A Natural Compound with Distinctive van der Waals Heterolayered Architecture

**DOI:** 10.1021/jacs.3c06433

**Published:** 2023-08-08

**Authors:** Luca Bindi, Xiuquan Zhou, Tianqi Deng, Zhi Li, Christopher Wolverton

**Affiliations:** † Dipartimento di Scienze della Terra, Università degli Studi di Firenze, Via G. La Pira 4, I-50121 Firenze, Italy; ‡ Materials Science Division, Argonne National Laboratory, 9700 South Cass Avenue, Lemont, Illinois 60439, United States; § State Key Laboratory of Silicon and Advanced Semiconductor Materials and School of Materials Science and Engineering, Zhejiang University, Hangzhou 310027, China; ∥ Institute of Advanced Semiconductors & Zhejiang Provincial Key Laboratory of Power Semiconductor Materials and Devices, ZJU-Hangzhou Global Scientific and Technological Innovation Center, Zhejiang University, Hangzhou 311215, China; ⊥ Department of Materials Science and Engineering, Northwestern University, Evanston, Illinois 60208, United States

## Abstract

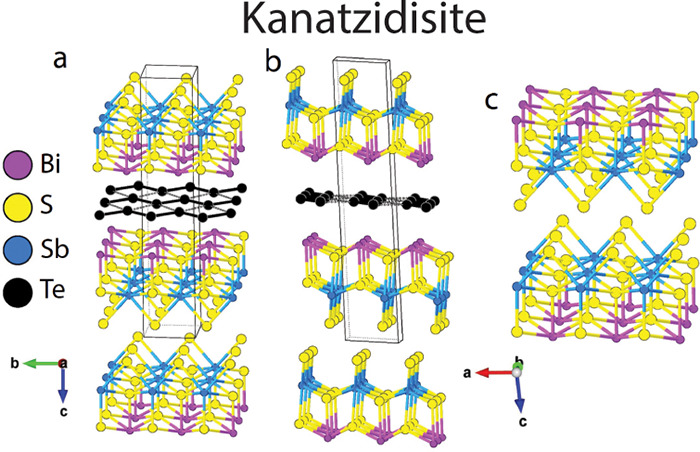

New minerals have long been a source of inspiration for the design and discovery. Many quantum materials, including superconductors, quantum spin liquids, and topological materials, have been unveiled through mineral samples with unusual structure types. In this report, we present kanatzidisite, a new naturally occurring material with formula [BiSbS_3_]_2_[Te_2_] and monoclinic symmetry (space group of *P*2_1_/*m*) with lattice parameters *a* = 4.0021(5) Å, *b* = 3.9963(5) Å, *c* = 21.1009(10) Å, and β = 95.392(3)°. The mineral exhibits a unique structure consisting of alternating BiSbS_3_ double van der Waals layers and distorted [Te] square nets essentially forming an array of parallel zigzag Te chains. Our theoretical calculations suggest that the band structure of kanatzidisite may exhibit topological features characteristic of a Dirac semimetal.

Naturally occurring minerals have a long history of inspiring the design and discovery of novel materials.^1^ For example, perovskite-related materials^2−4^ have been applied and engineered for various uses, and porous zeolites^5,6^ are widely used in the petroleum industry as catalysts. Some minerals exhibit rare and unimaginable structures instigating novel materials design, such as tochilinite,^7^ a mineral consisting of rare tetragonal anti-PbO type FeS and hexagonal brucite-type Mg(OH)_2_ heterolayers. Its structure inspired the design of high-temperature superconductors with critical transition temperatures (*T*_*c*_) of up to 43 K.^8,9^ Even compounds with new metal–organic frameworks (MOF) were discovered among minerals.^10^ The formation of minerals can also reveal synthetic conditions, such as when a lightning discharge over a dune in Nebraska created a novel quasicrystal of Mn_72.3_Si_15.6_Cr_9.7_Al_1.8_Ni_0.6_ with 12-fold symmetry.^11^ Therefore, to expand our materials database, especially when looking for inspiration for designing novel quantum materials, crystal structures of naturally occurring minerals can be convenient and robust sources^12^ for novel semiconductors,^13−18^ thermoelectrics,^19,20^ superconductors,^21−23^ quantum spin liquids,^24^ and topological materials.^25,26^ Sulfosalts make up a large group of minerals that exhibit a unique combination of metallic and chalcogen elements. They are typically characterized by a complex crystal structure and often contain multiple elements such as lead, silver, copper, and antimony.^27−30^ Sulfosalts are known for their diverse range of colors, from metallic gray to yellow, and for their semimetallic to opaque luster.^27,31^ Notable examples include bournonite,^32^ PbCuSbS_3_, also known as “cogwheel ore”, renowned for its distinctive twinned crystal structure, which forms interlocking crystal patterns that resemble the teeth of gears; tennantite,^33^ Cu_12_As_4_S_13_, adopting a crystal structure made by an unique three-dimensional arrangement of tetrahedra; and jamesonite,^34^ Pb_4_FeSb_6_S_14_, a complex compound with an orthorhombic crystal structure. Here we report a new sulfosalt mineral with the formula [BiSbS_3_]_2_[Te_2_]. It consists of rare distorted Te square nets and van der Waals BiSbS_3_ layers. The mineral has been approved by the International Mineralogical Association (IMA 2023–014) with the name kanatzidisite after the chemist Mercouri Kanatzidis, in honor of his achievements and innovations in chalcogenide chemistry.^35,36^

Kanatzidisite was detected in mining dumps of the abandoned Nagybörzsöny Au deposit at Alsó-Rózsa, northern Hungary. The mineralization is hosted by Miocene calcalkaline volcanic rocks and occurs as a stockwork in a propylitized dacite breccia pipe. The location and the material studied are the same as for jonassonite, Au(Bi,Pb)_5_S_4_,^37^ and jaszczakite, [(Bi,Pb)_3_S_3_][AuS_2_].^38^ Kanatzidisite occurs as a unique crystal with a black metallic luster. It is weakly bireflectant and weakly pleochroic from gray to a greenish gray. The mineral exhibits an anhedral grain morphology and does not show any inclusions of, or intergrowths with, other minerals. The maximum grain size of kanatzidisite is about 20 μm (Figure S1), and its composition was measured using wavelength dispersive spectrometers (WDS). The resulting empirical formula can be written as Sb_1.95_Bi_1.93_Pb_0.09_Au_0.01_S_5.94_Te_1.99_Se_0.09_ (Table S1). When excluding the small impurities of Au, Pb, and Se, the ideal formula is [BiSbS_3_]_2_[Te_2_], which is confirmed by the single crystal X-ray diffraction (SC-XRD) investigation (Tables 1 and S2).

**Table 1:** Crystal and Refinement Data for Kanatzidisite^a^

empirical formula	[BiSbS_3_]_2_[Te_2_]
crystal system	monoclinic
space group	*P*2_1_/*m*
crystal shape and color	metallic, black
volume (Å^3^)	335.99(6)
*Z*	2
density (g/cm^3^)	5.481
independent reflections	1641 [*R*_int_ = 0.0924]
data *k*/restraints/parameters	1641/0/37
goodness-of-fit	0.733
final *R* indices [*I* > 2σ(*I*)]	*R*_obs_ = 0.0276
	*wR*_obs_ = 0.0161
*R* indices [all data]	*R*_all_ = 0.0301
	*wR*_all_ = 0.0240

a A more detailed report of the crystal structure can be found in Table S2 and the CIF (CCDC 2270579).

Kanatzidisite is monoclinic, space group *P*2_1_/*m* (#11), with *a* = 4.0021(5) Å, *b* = 3.9963(5) Å, *c* = 21.1009(10) Å, and β = 95.392(3)° (Table 1). Powder X-ray diffraction data (PXRD) are listed in Table S3.

The structures of [BiSbS_3_]_2_[Te_2_] are shown in Figure 1a,b. The structure shows alternating “BiSbS_3_ double layers” and “[Te] layers” (Figure 1c). The double layers can be considered as composed of a pair of single BiSbS_3_ layers with ordered Bi and Sb atoms. Each BiSbS_3_ layer has a unique surface with sulfur atoms (doubly bridging with Sb atoms) on one side and a tetragonal Bi–S atom array on the other. When paired, these layers interact through the sulfur-containing sides *via* relatively long van der Waals distances (S–S) of 3.6216(16) and 3.9915(19) Å (Figure 2). These distances are slightly longer than the S–S distances in other van der Waals disulfides such as TiS_2_ (3.46 Å),^39^ MoS_2_ (3.49 Å),^40^ and SnS_2_ (3.61 Å).^41^ However, they are very similar to the S–S distances, 3.6455 and 3.8932 Å, between neighboring 1D van der Waals chains in Sb_2_S_3_.^42^ It should be noted that these are relatively long van Der Waals distances, and the existence of impurity atoms inserted in this space, such as the trace elements observed in the WDS analysis (Table S1), cannot be ruled out.

**Figure 1:**
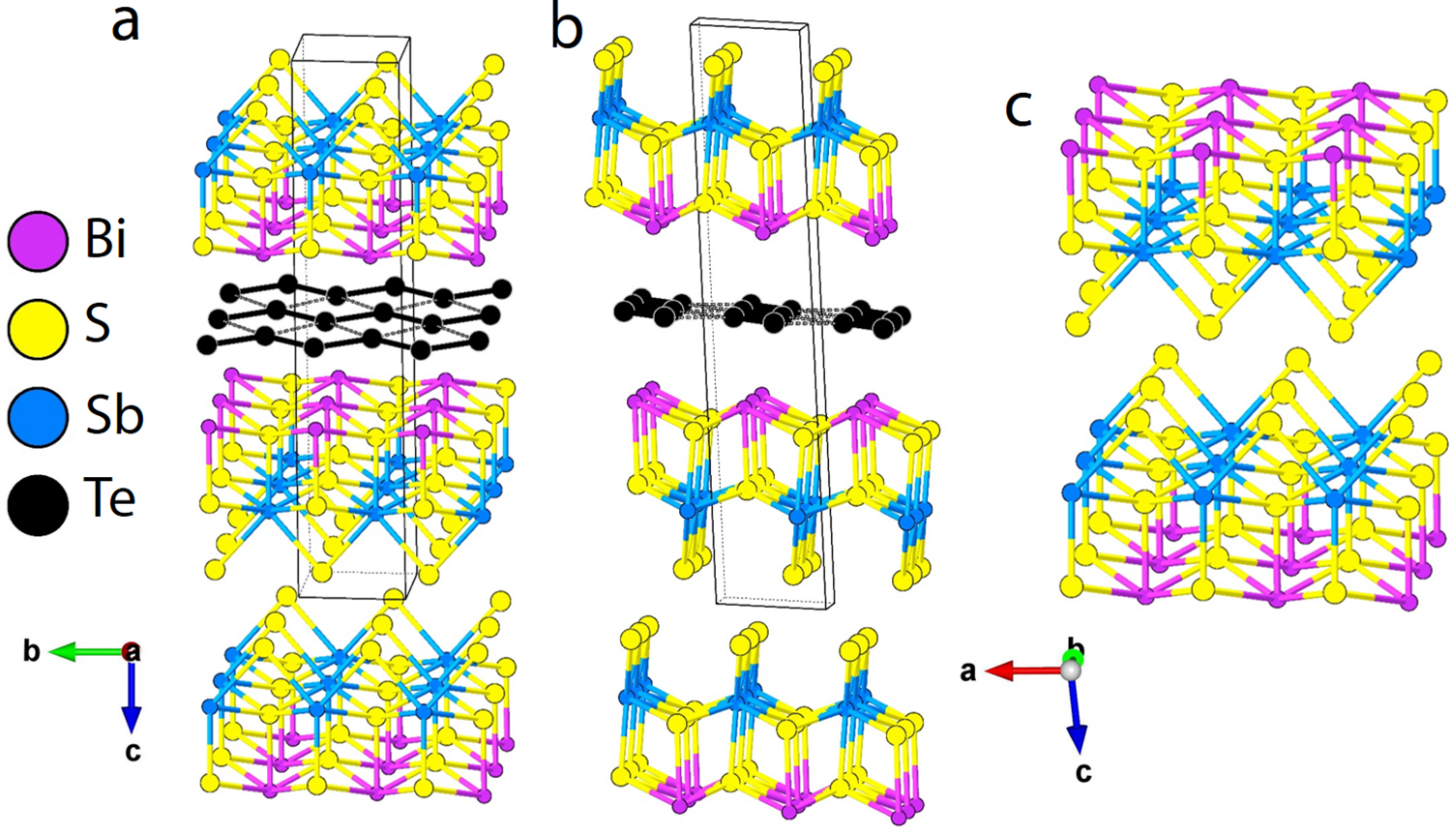
Crystal structure of [BiSbS_3_]_2_[Te_2_] along the a) *a*-axis, b) *b*-axis, and c) the BiSbS_3_ double layers. The overall structure is composed of alternating stacking of “BiSbS_3_ double layers” and “Te layers” along the *c*-axis.

**Figure 2:**
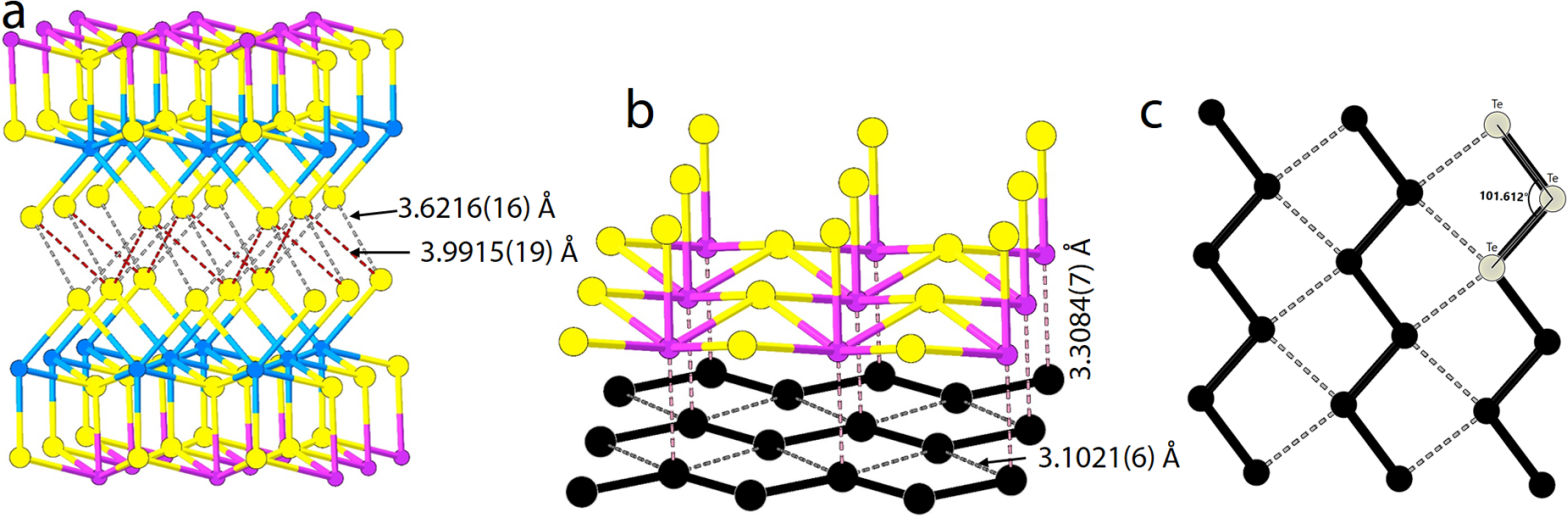
Bonding in [BiSbS_3_]_2_[Te_2_] showing a) the S–S bonds between the BiSbS_3_ double layer, b) distances between Te–Te in the Te square net, and c) bonds between Bi and Te.

The overall structure is composed of alternating stacking of “BiSbS_3_ double layers” and atomically thin “Te layers” along the *c*-axis and is connected by long Bi–Te bonds with a distance of 3.3084(7) Å (Figure 2a). The structure of the BiSbS_3_ double layers can be thought of as a biatomically thick slice out of the rock-salt structure-type cut along the [110] direction. The shortest and longest Bi–S and Sb–S bonds are 2.7248(6) and 3.1971(7) Å and 2.8887(7) and 3.0152(8) Å, respectively (Table 2). The Te layers are one atom thick with a square-net topology but are distorted, forming infinite zigzag chains running along the *b*-axis axis. The Te–Te bond length within the chains is 2.5782(5) Å, consistent with a single Te–Te bond. The [Te] chains are arranged in a parallel manner, with short interchain contacts measuring 3.1021(6) Å. This arrangement forms a distorted square-net structure that extends across the *ab*-crystallographic plane (Figure 2b). Taking the complete array of parallel chains into account, they collectively define a distorted square net made up of Te atoms. The Te–Te–Te angles within this structure are measured to be 101.61°. Researchers have proposed atomically thin layers of tellurium that have the potential to replace graphene in several electronics-related applications.^43^

**Table 2:** Selected Bond Distances (Å) for [BiSbS_3_]_2_[Te_2_] Obtained Using Single-Crystal Diffraction

Sb–	S1	3.0152(8) (×2)	Bi–	S2	3.0033(8)	Te-	Te–	2.5782(5) (×2)
	S 2	2.9016(9) (×4)		S3^1^	2.7248(6) (×2)		Te–	3.1021(6) (×2)
	S3	2.8887(7)		S3^2^	3.1971(7) (×2)			
S1–	S1^1^	3.6216(16) (×2)		Te	3.3084(7)			
	S 2^1^	3.9915(19) (×2)						

While the structure of the BiSbS_3_ layers in [BiSbS_3_]_2_[Te_2_] is unique, there are similarities with other compounds that have alternating blocks of metal sulfides composed of main-group elements (such as Bi, Sb, and S) and a monolayer of atoms arranged in an approximate square net (such as Te or Au/Te). Examples of such compounds include buckhornite ([Pb_3.1_Sb_0.9_S_4_][Au_*x*_Te_2-x_]),^22,44−46^ nagyagite ([Pb(Pb,Sb)_2_S_4_][(Au,Te)_2_])^47^ and jaszczakite, [(Bi,Pb)_3_S_3_][AuS_2_].^38^ Therefore, it is possible that trace amounts of Au or other atoms may also be present in the [BiSbS_3_]_2_[Te_2_] layers.

We carried out Density Functional Theory (DFT) calculations using the refined crystal structure obtained from SC-XRD as an initial guess. The relaxed crystal structure was subsequently submitted to the Open Quantum Materials Database (OQMD)^48^ to further verify its thermodynamic stability. Our DFT calculation resulted in a formation energy of −0.229 eV/atom, which is 0.169 eV/atom higher than the OQMD-predicted hull energy of −0.397 eV/atom (Figure S7). This suggests that kanatzidisite is metastable compared with a mixture of Bi_2_S_3_, Sb_2_S_3_, and Te. Hence, the formation of this mineral may not be easily accessible using direct reaction of elements or sulfides. The prevailing view suggests that sulfide minerals and sulfosalts are primarily formed through hydrothermal processes in nature^49,50^ and therefore emulating such conditions could be a promising avenue to pursue the laboratory synthesis of this new compound.

To capture the essential features of the electronic structure, we adopted the DFT-relaxed structure of impurity-free kanatzidisite for the static calculation, leaving the investigation of off-stoichiometry and impurity-related issues to future research. Figure 3a demonstrates that [BiSbS_3_]_2_[Te_2_], in the absence of spin–orbit coupling (SOC), exhibits the characteristics of a Dirac semimetal with a single Dirac point located along the Γ-Y path. With the inclusion of SOC, as observed in Figure 3b, noticeable band inversions occur along the Γ-Z, D-B, Γ-A, and Γ-Y paths, leading to localized band gaps at these points. However, the overall electronic structure of [BiSbS_3_]_2_[Te_2_] is semimetallic, with energetic overlaps between the conduction and valence bands, leading to a negative indirect gap. Further topological invariant analysis, including the parity analysis of the eight time-reversal-invariant moment (TRIM) points and the Wannier charge center calculations (Figure S8), confirms the nonzero *Z*_2_ invariant at the ***k***_3_ = 0.0 and ***k***_3_ = 0.5 planes (Figure S8e,f). These findings indicate that [BiSbS_3_]_2_[Te_2_] is a weak topological semimetal with a (ν_0_;ν_1_ν_2_ν_3_) configuration of (0;001). The right panel of Figure 3b illustrates the partial density of states (PDOS), indicating prominent contributions from the S 3p and Te 5p orbitals near the Fermi level. Additionally, Bi and Sb donate their 6p and 5s5p electrons to these chalcogen p orbitals, as further elucidated in the orbital-resolved DOS plots shown in Figure S2.

**Figure 3:**
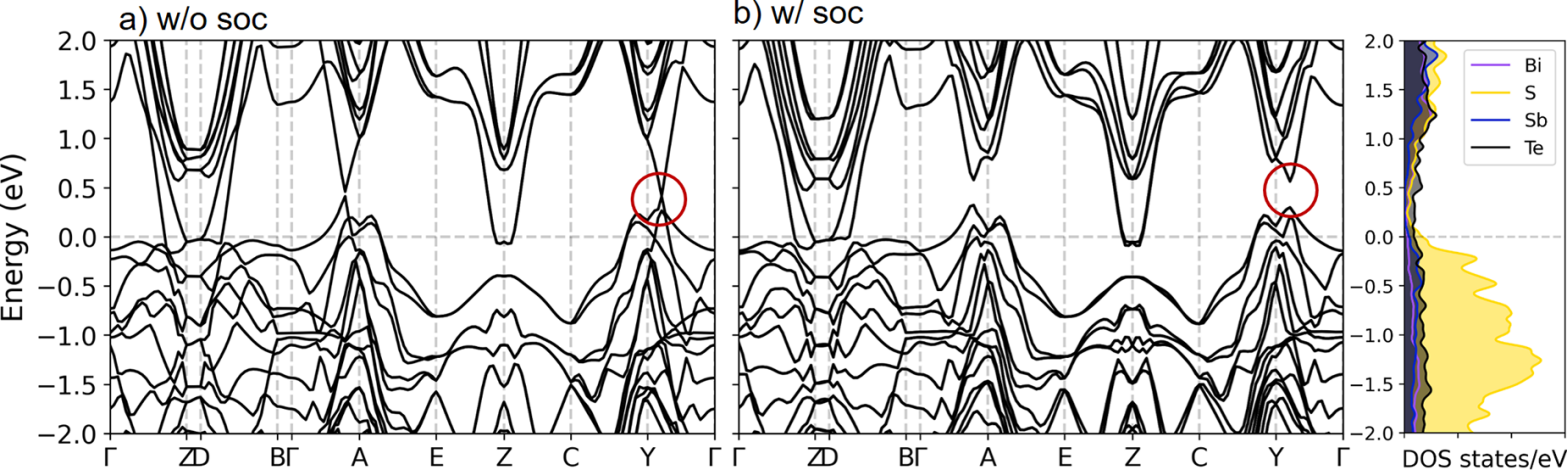
Band structures of kanatzidisite a) without spin–orbit coupling (SOC) and b) with SOC. The right panel of b) illustrates the partial density of states (PDOS) with SOC of c).

In order to enhance our insight into the bonding configurations in [BiSbS_3_]_2_[Te_2_], we analyzed the density of states (DOS) projected onto the band structure. Figures S3–S6 reveals an even distribution of Bi 6p, Sb 5p, and S 3p orbitals around the Fermi level throughout the entire Brillouin zone, indicating a conducting network within the [BiSbS_3_] layer. In the [Te] layers, the conduction and valence bands are predominantly dominated by Te p orbitals, implying a Te 5p–Te 5p hybridization within the plane. By projecting the density of states onto Te-centered Wannier functions and integrating the Te-partial density of states from the lowest energy level up to the Fermi level (Figure S9), we can accurately quantify the Te orbital filling. Our analysis reveals that exactly 12 valence electrons occupy Te-centered Wannier functions per unit cell, which corresponds to 6 electrons per Te atom. This observation suggests the existence of hypervalent bonding, originating from the half-filled p_*x*_/p_*y*_ orbitals of Te, as proposed by Papoian and Hoffmann,^51^ which rationalizes the formation of 2D square-net [Te] layers. Furthermore, Klemenz et al.^52^ proposed a tolerance factor *t* that predicts the occurrence of band inversion of half-filled p_*x*_/p_*y*_ orbitals and can be easily calculated from the distance between Te and neighboring atoms. For [BiSbS_3_]_2_[Te_2_], *t* is calculated to be 0.73, indicating the 2D nature of the Te layer and, more importantly, confirming the presence of band inversion (*t* ≤ 0.95). These results underscore that the semimetallic character of [BiSbS_3_]_2_[Te_2_] originates in the Te square nets.

In conclusion, we have discovered a new natural sulfosalt called kanatzidisite consisting of alternating slabs of BiSbS_3_ double layers and Te square nets. The discovery of this new mineral featuring alternating slabs of BiSbS_3_ double layers and Te square nets presents an exciting opportunity for materials design. This unique crystal structure, which exhibits a rare van der Waals heterolayer with Te square nets, serves as inspiration for the targeted synthesis of this and related novel materials with intriguing electronic properties, particularly in the realm of topological semimetals.^26,36,53^ While the exact electronic structure of kanatzidisite may differ from the calculated band structure due to the presence of minor elements like Au, Pb, and Se, similar to buckhornite and nagyagite, further investigations utilizing well-controlled chemical environments are necessary to fully understand the true nature and its potential.^46,47^

## Supporting Information Available

Characterization methods, topological invariant analysis, total and partial density of states, additional structural and chemical analyses data and SEM images (PDF)
