# Supplementary material for: Kanatzidisite: A Natural Compound with Distinctive van der Waals Heterolayered Architecture
Source: J Am Chem Soc. 2023 Aug 8;145(33):18227–32. doi: 10.1021/jacs.3c06433 (PMC10450802; doi:10.1021/jacs.3c06433)
Supplement: Supplementary file 1 — ja3c06433_si_001.pdf [file ja3c06433_si_001.pdf]

Kanatzidisite: A Natural Compound with Distinctive Van der Waals Heterolayered Architecture

Luca Bindi,[1*] Xiuquan Zhou,[2] Tianqi Deng,[3,4], Zhi Li[5], and Christopher Wolverton[3*]

1. Dipartimento di Scienze della Terra, Università degli Studi di Firenze, Via G. La Pira 4, I-50121 Firenze, Italy

2. Materials Science Division, Argonne National Laboratory, 9700 South Cass Avenue, Lemont, IL 60439, USA

3. State Key Laboratory of Silicon and Advanced Semiconductor Materials and School of Materials Science and Engineering, Zhejiang University, Hangzhou, 310027, China

4. Institute of Advanced Semiconductors & Zhejiang Provincial Key Laboratory of Power Semiconductor Materials and Devices, ZJU-Hangzhou Global Scientific and Technological Innovation Center, Zhejiang University, Hangzhou, 311215, China

5. Department of Materials Science and Engineering, Northwestern University, Evanston, Illinois 60208, USA

Experimental Details:

Characterizations. Single-crystal X-ray studies were carried out using a Bruker D8 diffractometer equipped with a Photon II CCD detector, with graphite-monochromatized Mo*K*α radiation (λ = 0.71073 Å). X-ray powder diffraction data were collected with an Oxford Diffraction Excalibur PX Ultra diffractometer fitted with a 165 mm diagonal Onyx CCD detector and using copper radiation (Cu*K*α, λ = 1.54138 Å). A preliminary chemical analysis using Energy Dispersive Spectroscopy (EDS) performed on the crystal fragment used for the structural study did not indicate the presence of elements (Z > 9) other than Sb, Bi, S and Te. However, given the minor elements found in jonassonite[1] and jaszczakite[2] coming from the same sample, we analyzed also Au, Ag, Pb, Cd, As, Se at the electron microprobe. Chemical analyses were carried out using a JEOL 8200 microprobe (WDS mode, 25 kV, 20 nA, 1 μm beam size, counting times 20 s for peak and 10 s for background). For the WDS analyses the following lines were used: Au*M*α, Ag*L*α, Bi*M*α, Pb*M*α, Cd*K*α, Sb*L*α, As*K*α, S*K*α, Se*L*α and Te*L*α.The crystal fragment was found to be homogeneous within analytical error.

Density Functional Theory Calculations. The Vienna ab initio simulation package (VASP)[3,4] implemented with projector augmented-wave method (PAW)[5] was used to perform all the density functional theory (DFT) calculations, including the geometry optimization, band structure, and density of states (DOS). We adopted a high cutoff energy of 500 eV of the plane-wave basis, which defines Bi 5d5s6p, Sb 5s5p, S 3s3p, and Te 5s5p electrons as valence electrons. The Perdew−Burke−Ernzerhof functional for solids (PBEsol)[6] was chosen as the exchange-correlation functional. When performing the geometry optimization, we utilized the experimental lattice parameters as the initial structure. The lattice size and shape as well as atomic coordinates were fully relaxed until the energy and force difference between the two self-consistent steps are less than 10^{-8} eV and 10^{-3} eV $Å^{-1}$. When calculating the band structure, we included the spin-orbital coupling (SOC) due to the presence of heavy elements Bi and Te.

Topological invariant analysis:

We performed parity analysis of wave functions in the eight time-reversal-invariant moment (TRIM) points (Fig. S7). We computed the δ_i quantity of each TRIM point Γ_i where we found that among the eight TRIM points, only $\Gamma_{000} = \mathbf{0}$ and $\Gamma_{001} = \frac{1}{2}\boldsymbol{b_3}$ has $\delta_i = -1$ while the other six

points exhibit $\delta_i = +1$. Therefore, according to the definitions by Fu and Kane,[7] Kanatzidisite is a weak topological semimetal with $(\nu_0; \nu_1\nu_2\nu_3) = (0; 001)$.

We further confirm the topological nature of Kanatzidisite via the Wilson loop (Wannier charge center) calculations on the six TRIM planes using WannierTools.[8] The Wannier basis was constructed using Wannier90 with 116 atomic-orbital-like spinor Wannier functions.[9] The Wilson loop calculations shows that only $\boldsymbol{k_3} = 0.0$ and $\boldsymbol{k_3} = 0.5$ planes exhibit non-zero Z_2 invariant, in agreement with the TRIM point calculations. Therefore, we can conclude that Kanatzidisite is a weak topological semimetal.

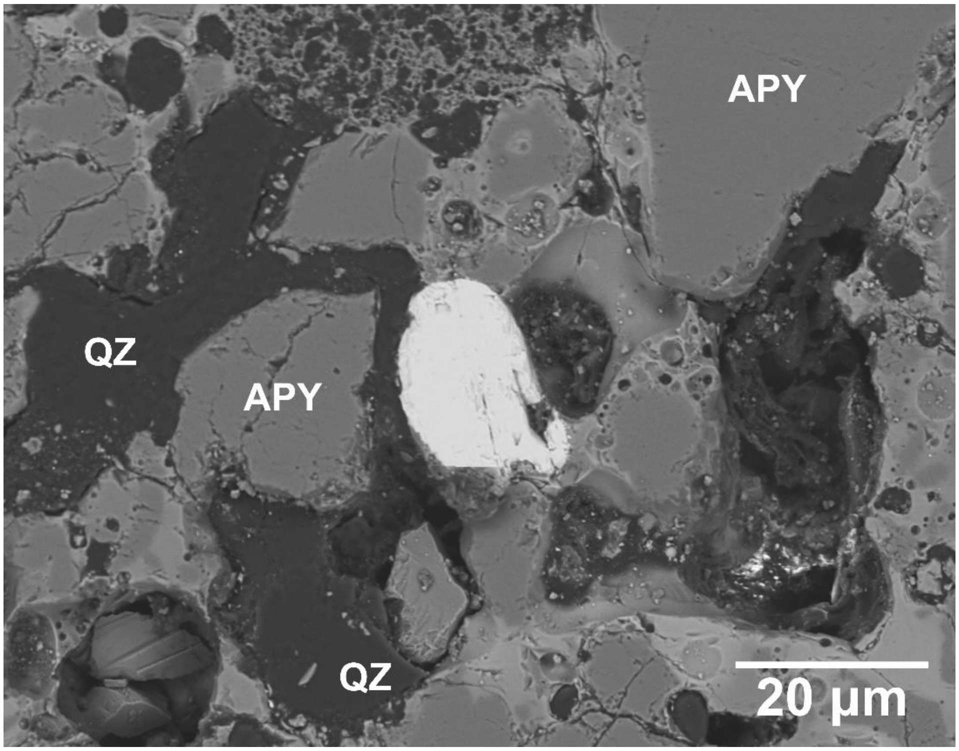

Figure S1. Scanning Electron Microscopy (SEM) image of kanatzidisite (bright grain in the middle) associated with arsenopyrite (APY) and quartz (QZ).

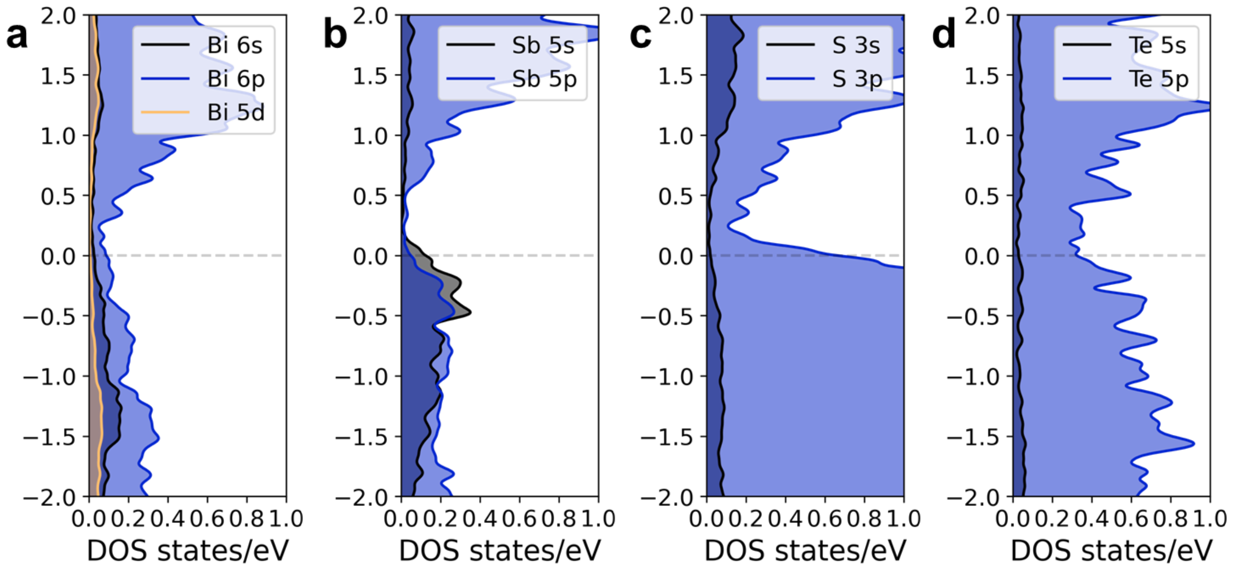

Figure S2. Orbital-resolved DOS for a) Bi, b) Sb, c) S, and d) Te

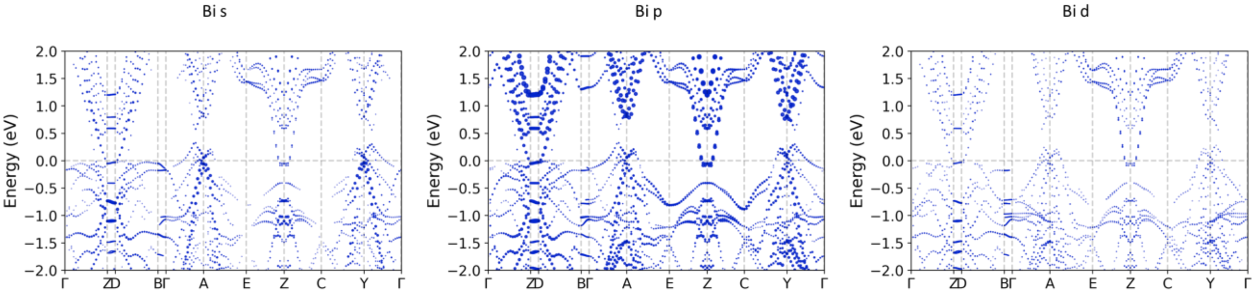

Figure S3. Orbital-projected band structure with spin-orbital coupling for Bi.

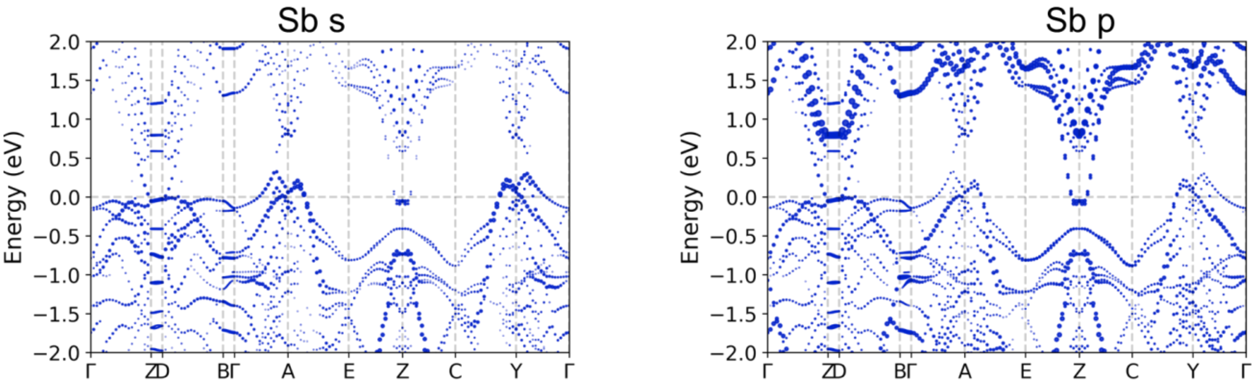

Figure S4. Orbital-projected band structure with spin-orbital coupling for Sb.

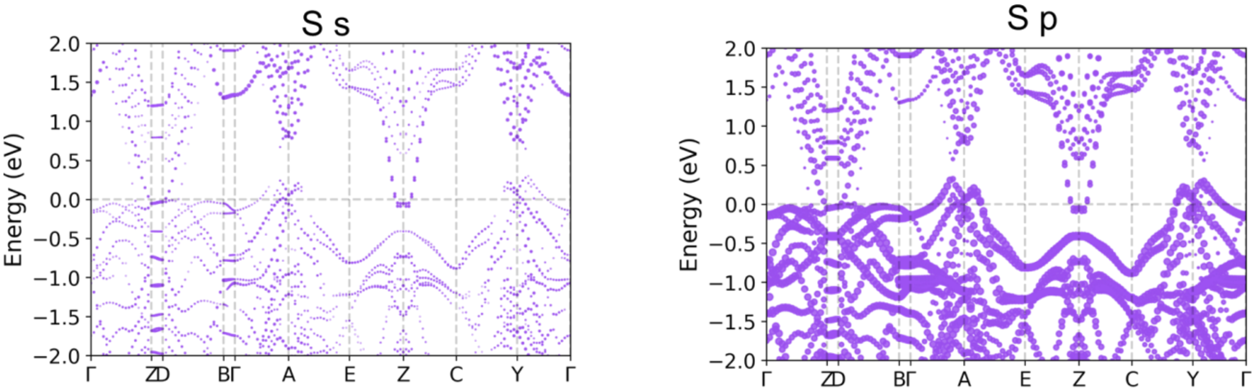

Figure S5. Orbital-projected band structure with spin-orbital coupling for Sb.

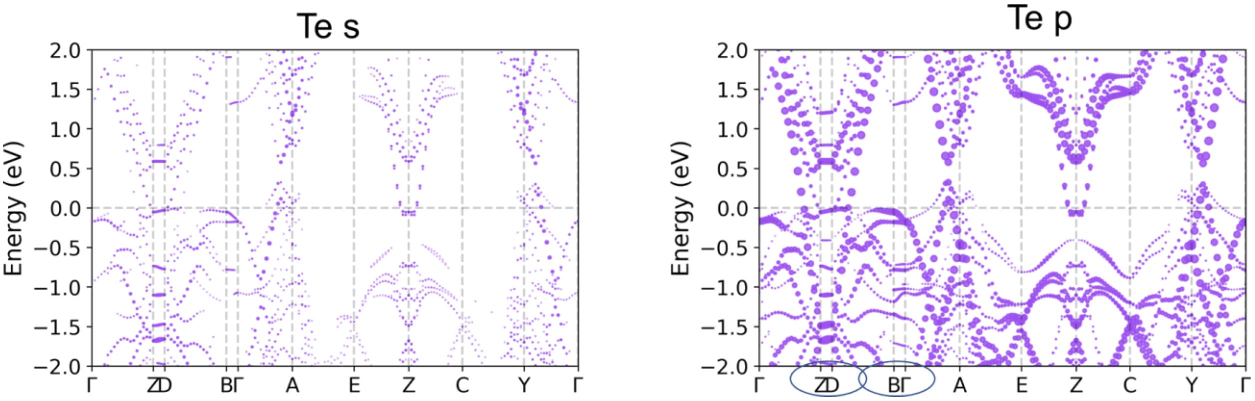

Figure S6. Orbital-projected band structure with spin-orbital coupling for Te.

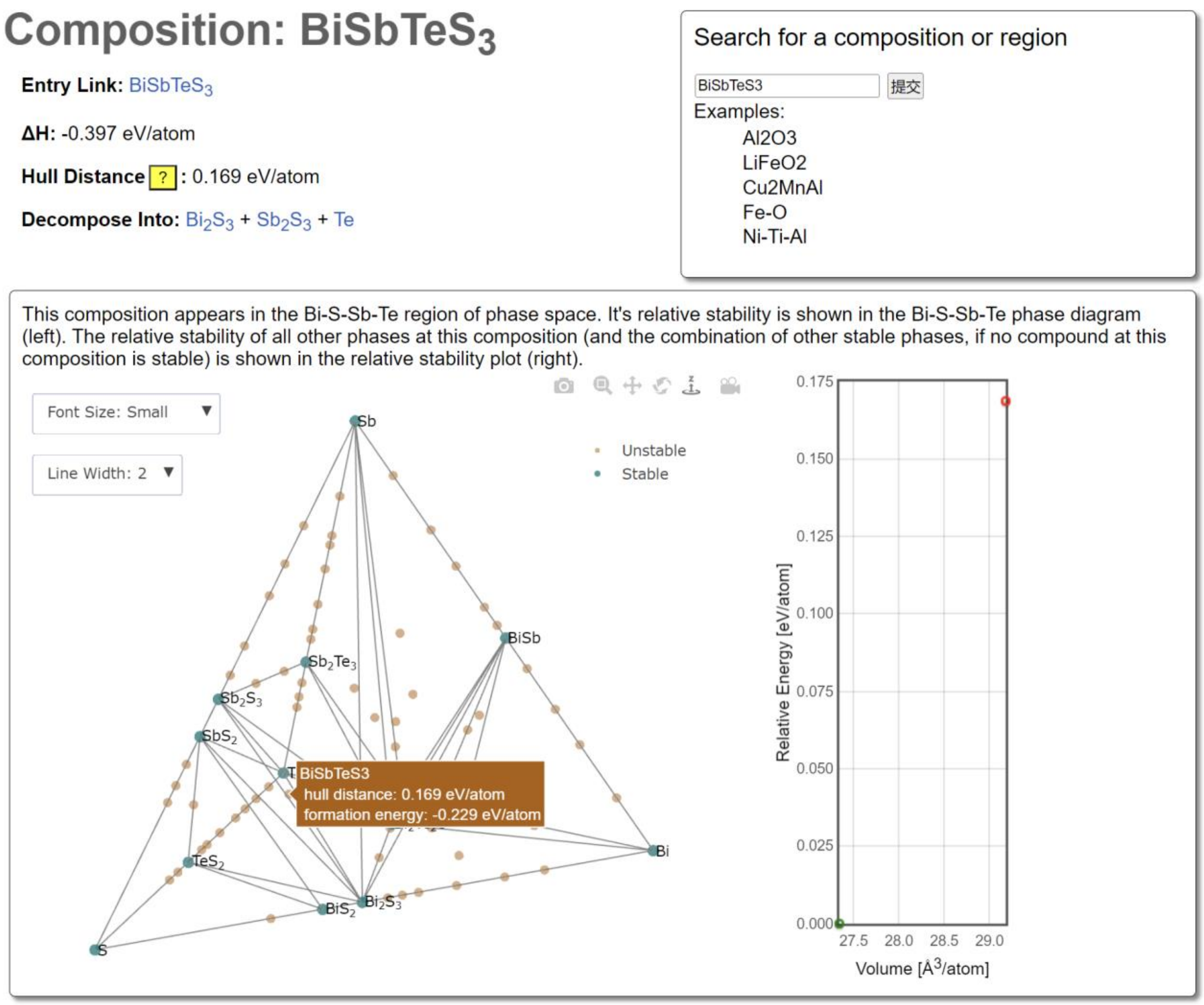

Figure S7. Formation energy of kanatzidisite compared to the convex hull obtained using the Open Quantum Materials Database (OQMD).[10]

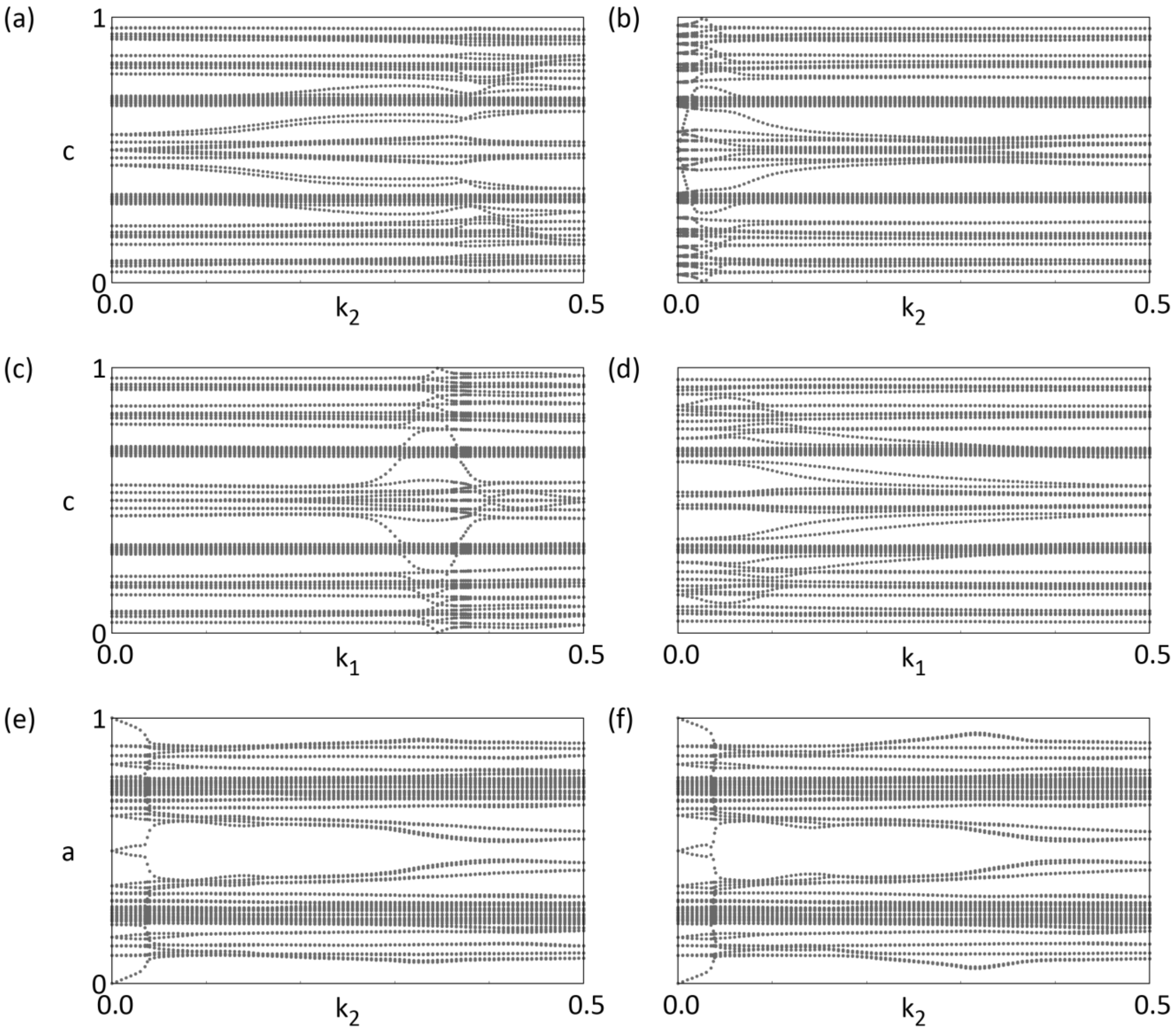

Figure S8. Wannier charge center evolution for the six time-reversal-invariant planes (a) $\boldsymbol{k_1} = 0.0$, (b) $\boldsymbol{k_1} = 0.5$, (c) $\boldsymbol{k_2} = 0.0$, (d) $\boldsymbol{k_2} = 0.5$, (e) $\boldsymbol{k_3} = 0.0$, and (f) $\boldsymbol{k_3} = 0.5$. Only the $\boldsymbol{k_3} = 0.0$ and $\boldsymbol{k_3} = 0.5$ planes exhibit non-zero Z_2 invariant.

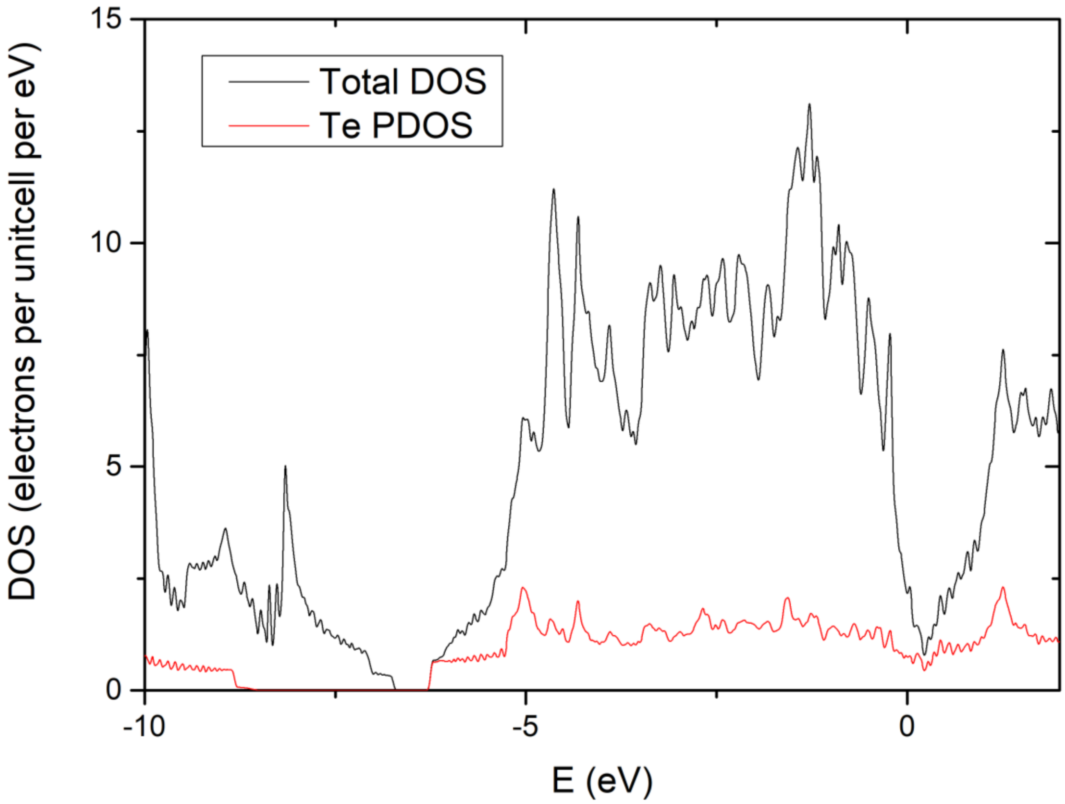

Figure S9. Total DOS and DOS projected onto Te-centered Wannier functions (Te PDOS). The Fermi level is the reference energy zero.

Table S1. Chemical analyses (in wt%) for kanatzidisite. The empirical formula (based on 12 atoms *pfu*) is $Sb_{1.95}Bi_{1.93}Pb_{0.09}Au_{0.01}S_{5.94}Te_{1.99}Se_{0.09}$, or, according to the structure refinement results $[(Sb_{1.95}Bi_{1.93}Pb_{0.09}Au_{0.01})_{\Sigma=3.98}(S_{5.94}Se_{0.09})_{\Sigma=6.13}][Te_{1.99}]$.The ideal formula is $[SbBiS_3]_2[Te_2]$, which requires Sb 21.96, Bi 37.68, S 17.35, Te 23.01, total 100.00 wt%.

Constituent	Mean	Range	SD	Probe Standard
Au	0.13	0.05 – 0.17	0.04	petzite
Ag	0.02	0.00 – 0.04	0.01	petzite
Bi	36.21	35.70 – 36.91	0.71	synthetic Bi_2S_3
Pb	1.63	1.45 – 1.88	0.11	galena
Cd	0.01	0.00 – 0.02	0.01	greenockite
Sb	21.37	21.04 – 21.78	0.32	synthetic Sb_2S_3
As	0.01	0.00 – 0.02	0.01	synthetic As_2S_3

S	17.09	16.50 – 17.61	0.27	galena
Se	0.63	0.41 – 0.90	0.05	clausthalite
Te	22.78	22.23 – 23.15	0.41	synthetic Sb_2Te_3
Total	99.87	99.41 – 100.42		

Table S2. Wyckoff positions, atom coordinates and equivalent isotropic displacement parameters (Å^2) for kanatzidisite.

atom	*Wyckoff*	*x*/*a*	*y*/*b*	*z*/*c*	U_{iso}
Sb	2*e*	0.16194(4)	¼	0.83258(2)	0.03023(4)
Bi	2*e*	0.35778(2)	¼	0.34482(2)	0.02521(3)
S1	2*e*	0.7365(3)	¼	0.06033(4)	0.05214(19)
S2	2*e*	0.3481(3)	¼	0.20233(3)	0.04814(18)
S3	2*e*	0.0747(2)	¼	0.69512(3)	0.04522(16)
Te	2*e*	0.29649(8)	¼	0.50009(2)	0.05632(6)

Table S2 extended. Anisotropic displacement parameters of the atoms for the selected kanatzidisite crystal

	U_{11}	U_{22}	U_{33}	U_{23}	U_{13}	U_{12}
Sb	0.03064(8)	0.03067(7)	0.02938(7)	0	0.00290(5)	0
Bi	0.02558(4)	0.02553(4)	0.02455(4)	0	0.00241(2)	0
S1	0.0526(5)	0.0518(5)	0.0520(4)	0	0.0048(3)	0
S2	0.0479(5)	0.0492(4)	0.0473(4)	0	0.0048(3)	0
S3	0.0456(4)	0.0459(4)	0.0442(3)	0	0.0044(2)	0
Te	0.05673(16)	0.05667(15)	0.05557(13)	0	0.00534(9)	0

Table S3. Observed and calculated X-ray powder diffraction data (*d* in Å) for kanatzidisite. 1 = calculated diffraction pattern obtained with the atom coordinates reported in Table 3 (only reflections with $I_{rel} \geq 5$ are listed); 2 = observed diffraction pattern.

			1		2	
h	*k*	*l*	d_{calc}	I_{calc}	d_{meas}	I_{meas}
0	0	2	10.5038	13	-	-
0	0	5	4.2015	9	-	-
1	0	0	3.9844	6	-	-
-1	0	1	3.9837	19	3.980	25
0	1	1	3.9259	8	-	-
-1	0	2	3.8472	48	3.850	55
0	1	2	3.7351	65	3.740	75
1	0	2	3.6144	24	3.615	20
-1	0	3	3.6120	8	-	-
0	0	6	3.5013	100	3.498	100
0	1	3	3.4709	6	-	-
1	0	3	3.3311	5	-	-
-1	0	4	3.3284	10	-	-
0	1	4	3.1803	38	3.178	45
0	0	7	3.0011	27		
1	1	0	2.8216	90	2.824	85
1	1	1	2.7722	88	2.770	80
-1	1	2	2.7716	8	-	-
1	0	5	2.7643	5	-	-
0	1	6	2.6335	14	-	-
1	0	6	2.5155	5	-	-
-1	0	7	2.5133	8	-	-
-1	1	5	2.4181	23	2.419	20
1	0	7	2.2957	9	-	-
-1	0	8	2.2939	21	-	-
-1	1	6	2.2721	63	2.270	50
0	1	8	2.1946	32	2.197	30
1	1	6	2.1289	31	2.125	25
-2	0	1	2.0010	6	-	-
0	2	0	1.9981	30	1.997	25
2	0	0	1.9922	5	-	-
1	1	7	1.9906	24	1.988	20
2	0	1	1.9660	13	-	-
1	0	9	1.9362	5	-	-
-2	0	5	1.8694	11	-	-
-2	1	1	1.7893	9	-	-
-1	2	2	1.7732	9	-	-
2	1	1	1.7641	5	-	-
0	0	12	1.7506	7	-	-

1	2	2	1.7487	5	-	-
0	2	6	1.7354	23	1.736	15
2	1	3	1.6941	5	-	-
0	2	7	1.6632	8	-	-
-2	1	7	1.5928	10	-	-
2	0	7	1.5923	7	-	-
-1	1	12	1.5340	12	-	-
-1	2	8	1.5067	9	-	-
2	1	7	1.4792	7	-	-
0	1	14	1.4048	7	-	-
2	2	1	1.4014	8	-	-
-2	2	5	1.3651	7	-	-
1	3	0	1.2634	6	-	-
1	3	1	1.2588	6	-	-
2	2	7	1.2453	5	-	-
-1	3	6	1.1998	6	-	-
2	1	13	1.1502	5	-	-

References:

1. Paar, W. H.; Putz, H.; Topa, D.; Roberts, A. C.; Stanley, C. J.; Culetto, F. J. Jonassonite, Au (Bi, Pb) 5S4, a new mineral species from Nagybörzsöny, Hungary. *The Canadian Mineralogist* **2006**, *44*, 1127.
2. Bindi, L.; Paar, W. H. Jaszczakite,[(Bi, Pb) 3S3][AuS2], a new mineral species from Nagybörzsöny, Hungary. *European Journal of Mineralogy* **2017**, *29*, 673.
3. Kresse, G.; Furthmüller, J. Efficient iterative schemes for ab initio total-energy calculations using a plane-wave basis set. *Physical Review B* **1996**, *54*, 11169.
4. Kresse, G.; Joubert, D. From ultrasoft pseudopotentials to the projector augmented-wave method. *Physical Review B* **1999**, *59*, 1758.
5. Blöchl, P. E. Projector augmented-wave method. *Physical Review B* **1994**, *50*, 17953.
6. Perdew, J. P.; Ruzsinszky, A.; Csonka, G. I.; Vydrov, O. A.; Scuseria, G. E.; Constantin, L. A.; Zhou, X.; Burke, K. Restoring the Density-Gradient Expansion for Exchange in Solids and Surfaces. *Physical Review Letters* **2008**, *100*, 136406.
7. Fu, L.; Kane, C. L. Topological insulators with inversion symmetry. *Physical Review B* **2007**, *76*, 045302.
8. Wu, Q.; Zhang, S.; Song, H.-F.; Troyer, M.; Soluyanov, A. A. WannierTools: An open-source software package for novel topological materials. *Computer Physics Communications* **2018**, *224*, 405.
9. Mostofi, A. A.; Yates, J. R.; Lee, Y.-S.; Souza, I.; Vanderbilt, D.; Marzari, N. wannier90: A tool for obtaining maximally-localised Wannier functions. *Computer physics communications* **2008**, *178*, 685.
10. Saal, J. E.; Kirklin, S.; Aykol, M.; Meredig, B.; Wolverton, C. Materials Design and Discovery with High-Throughput Density Functional Theory: The Open Quantum Materials Database (OQMD). *JOM* **2013**, *65*, 1501.
